# The effect of the PKR inhibitor, 2-aminopurine, on the replication of influenza A virus, and segment 8 mRNA splicing

**DOI:** 10.1515/biol-2025-1098

**Published:** 2025-04-28

**Authors:** Dagny Lorent, Rafal Nowak, Monika Gazecka, Pawel Zmora, Elzbieta Lenartowicz Onyekaa

**Affiliations:** Department of Molecular Virology, Institute of Bioorganic Chemistry Polish Academy of Sciences, 61-704, Poznan, Poland; Laboratory of Molecular Diagnostics, Institute of Bioorganic Chemistry Polish Academy of Sciences, 61-704, Poznan, Poland

**Keywords:** influenza A virus, mRNA splicing, pseudoknot structure, double-stranded RNA-activated protein kinase, non-structural proteins NS1 and NS2

## Abstract

Influenza A virus (IAV) is a negative-sense, single-stranded RNA virus, whose genome consists of eight segments encoding multiple structural and non-structural proteins essential for viral replication and host immune evasion. Among these, segment 8 encodes two primary non-structural proteins  NS1, crucial for host immune suppression and virulence and NS2, involved in vRNP export – and their expression is regulated by splicing. Further studies are required to elucidate the role of PKR in IAV mRNA splicing regulation.

## 1 Introduction

Influenza A virus (IAV) is a single-stranded, negative-sense RNA virus. Its small genome (around 13,500 bases) is divided into eight segments, which encode at least ten proteins: polymerase basic 2 (PB2), polymerase basic 1 (PB1), polymerase acidic (PA), hemagglutinin (HA), nucleoprotein (NP), neuraminidase (NA), matrix proteins 1 (M1) and 2 (M2), and non-structural proteins 1 (NS1) and 2 (NS2) [1,2,3]. Eight proteins, i.e., PB2, PB1, PA, HA, NP, NA, M1, and M2, are crucial and structural. IAV also expresses non-structural proteins (e.g., NS1, NS2, PA-X, PB1 frame 2 [PB1-F2], PB1-N40, PA-N155, PA-N182, M42, and NS3), which can play a role in host defense suppression, virulence, and pathogenicity [1–3].

Different viral mRNAs can be generated through splicing, frameshift, or truncation of the coding region of the viral gene [4]. However, the detailed mechanism of IAV splicing is still unknown, and there is no evidence showing how the IAV regulates the amount of spliced mRNA. However, it is known that the splicing of IAV can appear without viral protein. This means that the IAV hijacks cellular machinery; therefore, the entire splicing process can be regulated by host factors [5].

Double-stranded RNA (dsRNA)-activated protein kinase R (PKR) is usually considered as a crucial player in the immune response to virus infection, since it senses dsRNA, is often a product of virus replication [6]. Activation of PKR by dsRNA leads to the phosphorylation of eukaryotic initiation factor-2α (eIF2α), which results in the shutdown of cellular and viral protein synthesis in cells and inhibition of virus spread [7]. The effective PKR activation usually requires a double-helical RNA structure with minimal and optimal size of 16–18 base pair (bp) and 40–80 bp, respectively. Recently, it was found that some short elements within mammalian mRNA, such as interferon-γ (IFN-γ) 5′-proximal pseudoknot and tumor necrosis factor α (TNF-α) 3′-untranslated region, which also folds into a pseudoknot, may activate PKR and control its mRNA splicing [8,9]. Whether other pseudoknot structures among different mRNAs may affect their splicing through the PKR activation remains an open question.

**Figure 1:**
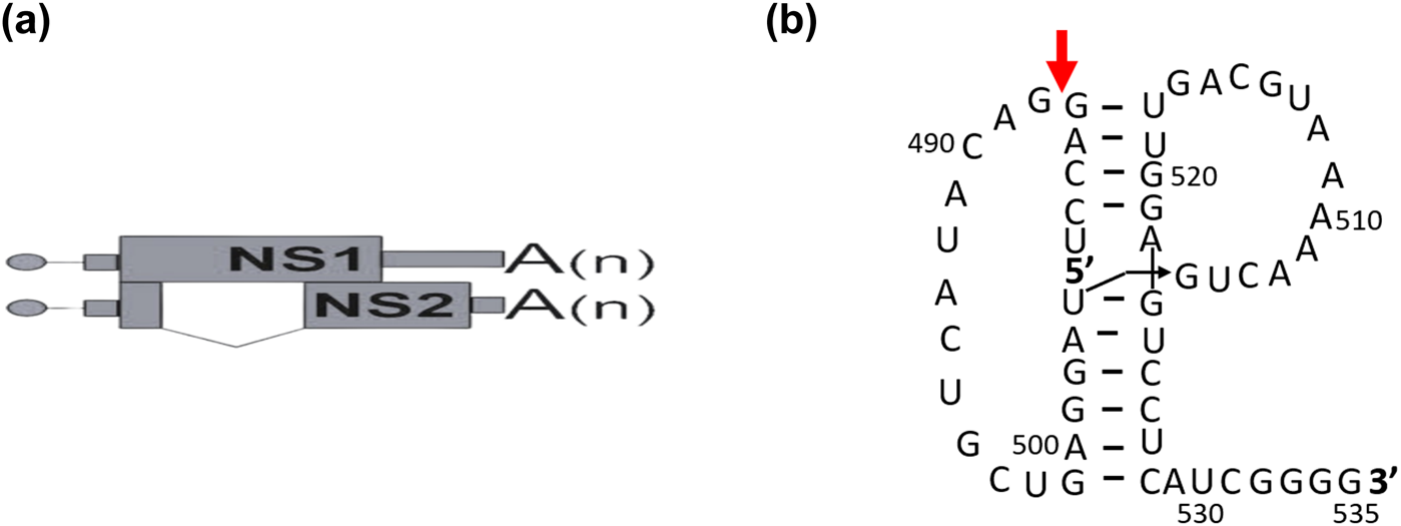
(a) Schematic presentation of NS1 and NS2 mRNA. A dot indicates a 5′ capp, the small blocks show the untranslated regions, and bigger boxes the coding region for each protein, line – intron, and A(n) – poly-A tail. (b) Structure of the NS1 mRNA fragment, which can fold into a pseudoknot. (b) is done based on the study of Jiang et al. [14].

This study focuses on IAV segment 8, which encodes two main proteins (NS1 and NS2) (Figure 1) [10]. NS1 is translated from non-spliced mRNA and plays an essential role in interaction with host factors. For example, it is important in the suppression of host innate immunity and in shutting off host gene expression, virulence, or apoptosis [11]. NS2 mRNA is spliced from the mRNA of full-length segment 8 and is also known as nuclear export protein (NEP). NEP plays an important role in vRNP transport from the nucleus to the cytoplasm and some studies have indicated that the NS2 protein impacts IAV transcription and replication [12,13]. Simultaneously, structural studies indicated a pseudoknot/hairpin structure equilibrium at the 3′ splice site in segment 8, whose biological function thus far remains unknown (Figure 1) [14].

Here, using 2-aminopurine (2-AP), a PKR inhibitor, we wanted to check whether the pseudoknot in the IAV segment 8 structure can interact with PKR to regulate viral mRNA splicing.

## 2 Materials and methods

### 2.1 Cells and viruses

The HEK-293T (CRL-3216, ATCC), MDCK 2 (CRL-2936, ATCC), and A549 (CCL-185, ATCC) cells were grown at 37°C in a 5% CO_2_ humidified incubator in Dulbecco’s modified Eagle’s medium (DMEM; Gibco, Thermo Scientific, Waltham, MA, USA) supplemented with 10% fetal bovine serum (Gibco, Thermo Scientific, Waltham, MA, USA) and PS (100 U/mL of penicillin and 100 μg/mL of streptomycin; Gibco, Thermo Scientific, Waltham, MA, USA). The influenza virus A/PR/8/34 (H1N1) (PR8) was reconstituted with an 8-plasmid system and the viral titer was measured with focus forming assays, as described previously [15].

### 2.2 Cell viability assay

For the cell viability assays, the HEK-293T, MDCK 2, and A549 cells were seeded at 96-well plated at a density of 2 × 10^5^ cells/mL and incubated overnight at 37°C and 5% CO_2_. After 24 h cells were washed with PBS, a fresh culture medium containing 5, 2.5, or 0.5 mM of 2-AP was added, and cells were further incubated for 24 h. The control (mock-treated) cells were incubated with the appropriate volume of the solvent, i.e., PBS. Next, the cell viability was measured using the CellTiter-Glo^®^ Assay (Promega, Madison, Wisconsin, USA) according to the manufacturer’s recommendations. Briefly, the culture medium was carefully removed, cells were incubated with 50 μL of reagent for 10 min, and next transferred to 96-well white plates to measure the luminescence with Hidex Sense Plate Reader (Hidex, Turku, Finland).

### 2.3 IAV infection experiments

The HEK-293T, MDCK 2, and A549 cells were seeded in 24-well plates at a density of 2.5 × 10^5^ cells/well. After 24 h, the cells were gently washed with PBS and next infected with influenza virus A/PuertoRico/8/34 (H1N1) (IAV PR8) at an MOI 0.01 in the infection medium (DMEM supplemented with 0.2% BSA, 100 U/mL of penicillin, and 100 μg/mL of streptomycin). After 1 h of incubation, the infection medium was removed and a fresh infection medium with 2-AP (Merck, Darmstadt, Germany) at a final concentration of 5, 2.5, or 0.5 mM was added. Additionally, the infection medium for the HEK-293T, MDCK 2, and A549 cells was supplemented with the 2.5 μg/mL N-acetylated trypsin (Merc, Darmstadt, Germany). Supernatants and cells were collected at 24 h post-infection for further analysis.

### 2.4 IAV replication analysis

The viral RNA was isolated from cell-free supernatants of the above-mentioned IAV PR8 infected and 2-AP treated cells with NucleoSpin RNA Virus Kit (Macherey Nagel, Duren, Germany) according to the manufacturer’s protocol. For the determination of viral RNA amount, the qRT-PCR was carried out using a TaqPath™ 1-Step RT-qPCR Master Mix, CG reagent kit with gene-specific primers (5′-GTTGGTAATGAAACGGAAACG-3′; 5′-GGCCATCCGAATTCTTTTG-3′) and probe (5′-TGACAGCCAGACAGCGA-3′). The PCR protocol consists of 2 minuracil-N glycosylase incubation at 25°C, reverse transcription for 15 min at 50°C, initial denaturation at 95°C for 2 min, and 40 cycles of 95°C for 15 s and 60°C for 60 s. The reaction was performed on the BIO-RAD CFX96 Real-Time system.

To define the limit of detection and to generate a standard curve to calculate the absolute copy number from the cycle threshold value, the specific standards were made as 10-fold dilutions of RNA transcribed on an IAV template. Six serial dilutions starting from 3.6 × 10^7^ total number of viral RNA genome copies were used.

### 2.5 NS1 and NS2 mRNA splicing analysis

To analyze the impact of 2-AP on the expression level of IAV NS1 and NS2, the total RNA from IAV infected and 2-AP treated cells were isolated with NucleoSpin RNA Kit (Macherey Nagel, Duren, Germany) according to the manufacturer’s protocol. Furthermore, we reverse-transcribed the isolated RNA with SuperScript^®^ IV Reverse Transcriptase (Invitrogen, Thermo Scientific, Waltham, MA, USA) and oligo dT according to the manufacturer’s protocol. For the determination of viral mRNA NS1 and NS2 RNA expression, the qRT-PCR was carried out using PowerUp™ SYBR™ Green Master Mix and primers for NS1: 5′-CAGCACTCTTGGTCTGGACA-3′, 5′-TGGACCATTCCCTTGACATT-3′ and for NS2: 5′-GCTTTCAGGACATACTGCTGAG-3′, 5′-CGCCATTTCTCGTTTCTCTT-3′, respectively. The PCR protocol consists of 2 min uracil-DNA glycosylases activation at 50°C, denaturation at 95°C for 2 min, and 40 cycles of 95°C for 15 s and 60°C for 60 s. The reaction was performed on the BIO-RAD CFX96 Real-Time system.

To generate a standard curve for NS1 and NS2 mRNA quantification, the specific standards were made as 10-fold dilutions of NS1, NS2, and actin DNA. Six serial dilutions starting from concentration 6 × 10^−6^ ng/mL for NS1 DNA, 1.5 × 10^−6^ ng/mL for NS2 DNA, and 1.4 × 10^−6^ ng/mL for actin DNA were used. The general NS1 and NS2 gene expression was calculated as starting quantity (SQ) with actin as a housekeeping gene.

### 2.6 Western blot analysis

To analyze the impact of 2-AP on the IAV NS1 and NS2 proteins expression, the HEK-293T, MDCK 2, and A549 cells were infected and treated with 2-AP as described above. Next, whole virus-cell lysates were prepared at 24 h post-infection using a lysis buffer (60 mM Tris pH 6.8, 10% glycerol, 2% SDS, 5% β-mercaptoethanol, 0.1% bromophenol blue, and 1 mM EDTA) and heated to 90°C for 5 min. The resulting protein lysates were separated on a 4–15% Mini-PROTEAN TGX Stain-Free Protein Gels (Bio-Rad) and then transferred to a nitrocellulose membrane with Trans-Blot Turbo Transfer System (Bio-Rad, USA). To confirm the 2-AP inhibitory effect on PKR activity, we analyzed the phosphorylation of translation initiation factor eIF2α mediated by PKR with rabbit monoclonal antibody specific for p-eIF2α (phospho-eIF2α (Ser51) antibody, 701268, Invitrogen). The NS1 and NS2 protein expression was detected using rabbit polyclonal antibodies specific for each protein, i.e., PA5-23365 for IAV NS1 and PA5-32234 for IAV NS2 (Thermo Scientific, Waltham, MA, USA) in conjunction with HRP-coupled goat anti-rabbit IgG antibody (Merck, Darmstadt, Germany). Expression of actin, which served as a loading control, was detected using mouse monoclonal antibody to actin (ACTN05(04); Invitrogen, Thermo Scientific, Waltham, MA, USA) in conjunction with HRP-coupled goat anti-mouse IgG antibody (Merck). Chemiluminescence signals were detected with the WESTAR NOVA 2.0 system (Cyanagen, Bologna, Italy) according to the manufacturer’s instruction and visualized using an Azure 400 fluorescent imager (Azure Biosystems, USA).

### 2.7 Statistical analysis

One- or two- way ANOVA with the Tukey *post-hoc* test was used to test statistical significance of data originating from single representative experiments performed in triplicate and independently repeated at least three times. The statistical differences between tested groups are marked with A, B, C, D, E. All statistical analyses were performed using GraphPad Prism 9 software.

## 3 Results

### 3.1 Effect of 2-AP on cell viability

Although 2-AP is a known PKR inhibitor, we checked cell viability in the presence of a small molecule according to the manufacturing protocol. No significant differences were noticed in cell propagation in any of the 2-AP concentrations used (data not shown).

### 3.2 Effect of 2-AP on IAV replication

**Figure 2:**
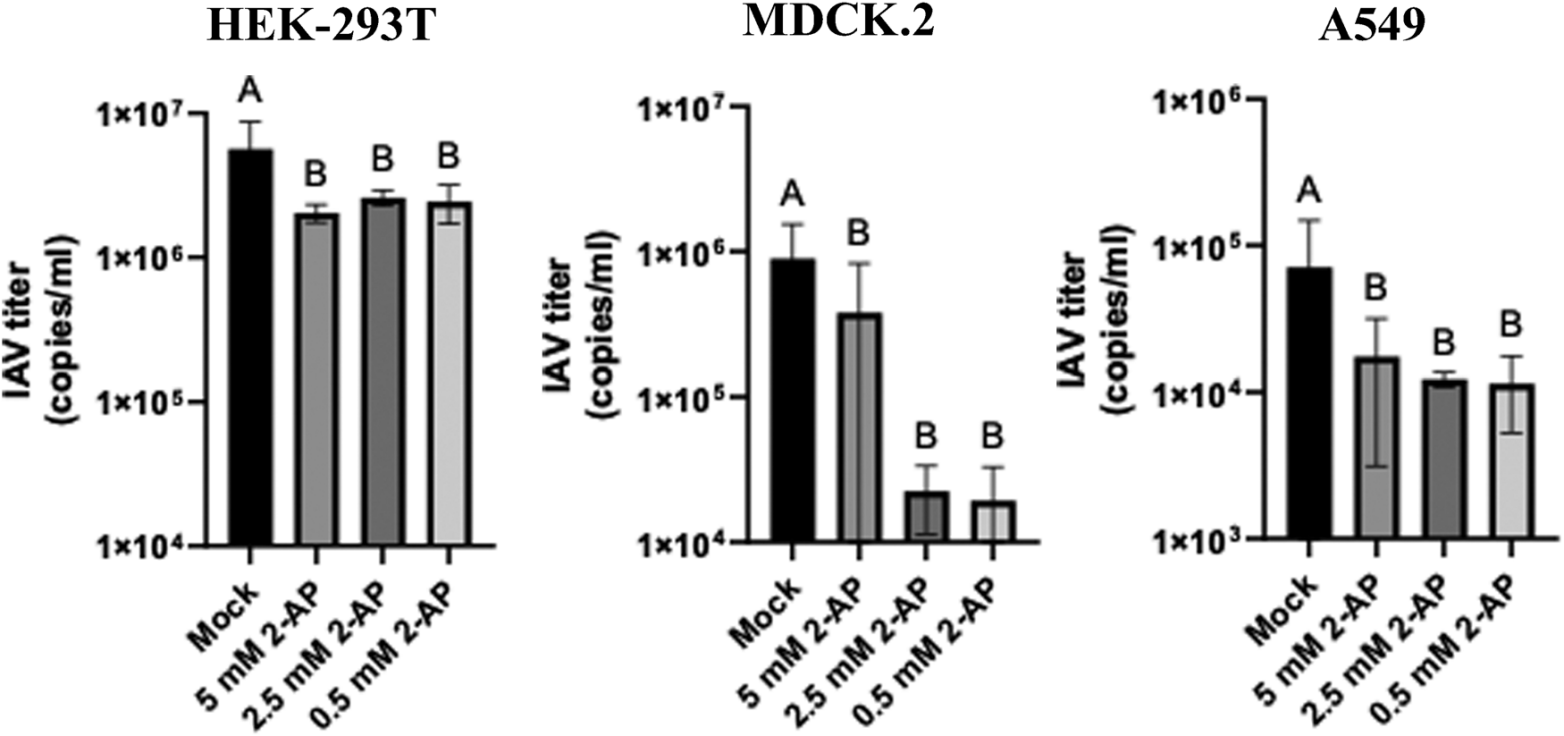
IAV replication in cells treated with 2-AP. HEK-293T (left panel), MDCK 2 (middle panel), and A549 (right panel) were infected with influenza virus A/PuertoRico/8/34 at an MOI 0.01 and treated with different 2-AP amounts, i.e., 5, 2.5, and 0.5 mM. The copy number of viral RNA in supernatant was determined by RT qPCR at 24 h post-treatment. The results of representative experiments performed with triplicate samples are shown. Error bars indicate standard deviations (SD). Similar results were obtained in three independent experiments. A, B – values with different superscript letters differ *p* < 0.05.

2-AP was tested in three concentrations (5, 2.5, and 0.5 mM) in three cell lines (HEK-293T, MDCK 2, and A549) infected with IAV PR8 at an MOI 0.01. The viral titer in cell culture supernatant at 24 h post infection differed between cell lines, i.e., HEK-293T cells produced 1- and 2-log more infectious viral particles than MDCK 2 and A549 cells, respectively (Figure 2). Simultaneously we observed that the IAV titer was significantly lower in each cell line treated with 2-AP, with the highest inhibitory effect found in cells with moderate IAV replication, i.e., MDCK 2 and A549, by about 2 logs and 1 log difference between mock-treated cells and cells incubated with 2-AP, respectively. On the other hand, the lowest inhibitory effect of 2-AP was detected in HEK-293T cells (up to 0.5 log), which were found to generate highest number of IAV virions (Figure 2). Interestingly, we did not observe any significant effect of 2-AP dose on IAV titer, this confirms how potent inhibitor is the 2-AP and that small dose is enough to inhibit PKR activity. Finally, our results suggest that the effect of 2-AP is cell type dependent and may be related to the IAV replication scale.

### 3.3 Effect of 2-AP on NS1 and NS2 mRNA transcription and splicing

**Figure 3:**
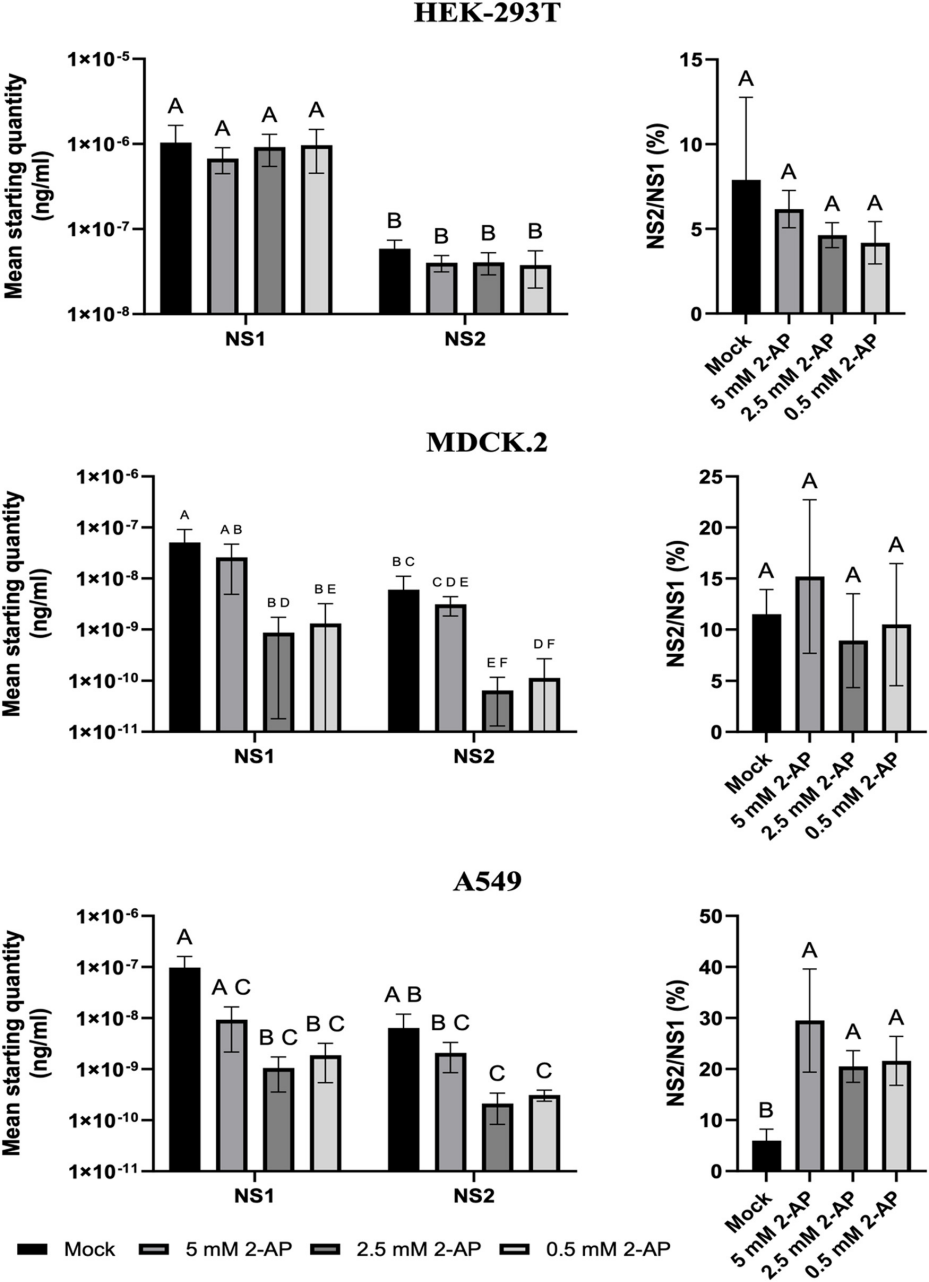
NS1 and NS2 expression (left panel), as well as NS2/NS1 ratio (right panel), in cells treated with 2-AP. HEK-293T, MDCK 2, and A549 were infected with influenza virus A/PuertoRico/8/34 at an MOI 0.01 and treated with different 2-AP amounts, i.e., 5, 2.5, and 0.5 mM. Concentration of NS1 and NS2 in cell lysates was determined by RT qPCR (left panel). The NS2/NS1 ratio was calculated based on the mean SQ. The results of representative experiments performed with triplicate samples are shown. Error bars indicate SD. Similar results were obtained in three independent experiments. A, B – values with different superscript letters differ *p* < 0.05.

The effect of 2-AP on segment 8 RNA transcription and splicing was checked using real-time qPCR. In general, the NS1 was significantly highly expressed than NS2 in HEK-293T and MDCK 2 cells, while no significant difference in NS1 and NS2 expression was found in A549 cells (Figure 3). This suggests that the IAV mRNA transcription and splicing depends on the cell types and may vary between them, and thus the PKR inhibition may have dramatically different results. For example, we did not observe any significant effect of 2-AP on both, NS1 and NS2, transcript levels in HEK-293T cells (Figure 3, left panel), and this explains the lowest inhibitory effect of 2-AP on IAV titer in this cell line (Figure 2). A statistically significant decrease in the concentrations of mRNA NS1 and NS2 was observed in MDCK 2 cells treated with 2-AP, as well as in A549 cells (Figure 3, left panel). This finding aligns with the previously reported lower titers of IAV in the cell culture treated with 2-AP (Figure 2). Also, similar to the experiments on the effect of 2-AP on IAV titer, we did not notice any significant 2-AP dose-dependency on the NS1 and NS2 expression.

The NS2/NS1 ratio was determined for each 2-AP concentration and for the control (Figure 3, right panel). There were no significant differences in the mRNA ratios between the control group and the cells treated with 2-AP in either MDCK 2 or HEK-293T cells. A significant difference was observed for A549 cells. In the 2-AP-treated cells, the amount of mRNA NS2 was significantly higher than in the mock-treated cells. Similar to previous experiments, the 2-AP amount used for cell treatment did not have any significant effect on NS2/NS1 ratio.

**Figure 4:**
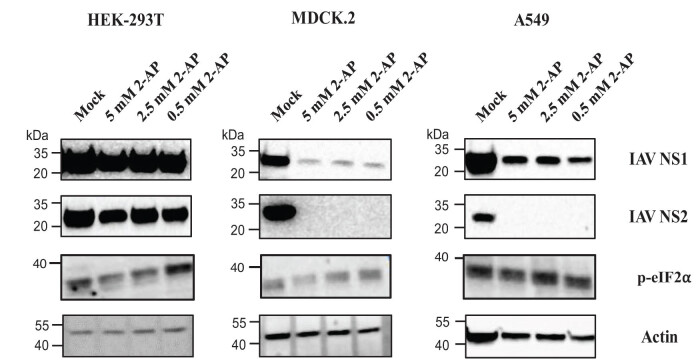
Inhibition of PKR activity and its effect on expression of IAV NS1 and NS2 in cells treated with 2-AP. HEK-293T (right panel), MDCK 2 (middle panel), and A549 (right panel) were infected with influenza virus A/PuertoRico/8/34 at an MOI 0.01 and treated with different 2-AP amounts, i.e., 5, 2.5, and 0.5 mM. The inhibition of PKR was demonstrated by the expression of p-eIF2α, a factor which was shown to be phosphorylated by PKR. The expression of NS1 and NS2 in the cell lysates was visualized with specific antibodies. The results of representative experiments are shown. Similar results were obtained in two independent experiments.

Subsequently, real-time PCR results were confirmed by western blot analysis. First, to confirm the 2-AP inhibitory effect on PKR, we checked direct PKR activity measurements, showed as an expression of phosphorylated eIF2α (p-eIF2α). In HEK-293T and MDCK 2 cells, 2-AP treatment caused dose-dependent inhibition of PKR activity and showed a lower expression of p-eIF2α on the western blot. Interestingly, the PKR activity in A549 cells was significantly reduced only when the highest 2-AP concentration was used, which suggests that the amount of PKR inhibitor needed for the inhibition of IAV replication is lower than for stopping PKR activity itself. With specific antibodies, we detected the NS1 and NS2 proteins in the cell lysate from all analyzed IAV-infected mock-treated cells. Upon 2-AP treatment, expression of NS1 and NS2 proteins was not changed only in HEK-293T cells (Figure 4), as it was shown in the real time PCR results. Also, in agreement with results presented above, NS1 protein expression was detected in MDCK 2 and A549 cells treated with 2-AP, but at a significantly lower level. On the other hand, we did not observe any expression of NS2 protein upon addition of the PKR inhibitor, which may be explained by extremely low level of protein expression, as shown by the real-time PCR results, which is almost impossible to be visualized with western blotting.

## 4 Discussion

During a viral infection, dsRNAs are generated and may activate the PKR, which subsequently leads to the phosphorylation of eIFα and to mRNA translation inhibition [16]. PKR not only inhibits translation in the cytoplasm but is also presented in the nucleus and can regulate pre-mRNA splicing of TNF-α, globin, or IFN-γ [8,9,17,18]. For example, the efficient splicing of TNF-α pre-mRNA is associated with a 104-nt 2-aminopurine response element (2-APRE), located in 3′-UTR. It was shown that 2-APRE can activate PKR more effectively than dsRNA. PKR requires at least 16–18 bp to sufficiently bind to dsRNAs, but its activation requires at least 33 bp and optimal 80 bp fragments. Simultaneously, internal bulges and non-Watson-Crick structures in RNA are tolerated during PKR binding. In the case of TNF-α, the pseudoknot structure is essential as an autophosphorylation activator of PKR. Two double helices that form the pseudoknot stack with parallel axes make the helix long enough to bind a PKR monomer, which facilitates kinase dimerization and activation [9,18].

Here, we wanted to check whether the pseudoknots in the IAV segment 8 mRNA can interact with PKR and be used as a splicing regulator of virus mRNAs. Our inquiry is based on the observations made by Jiang et al. and Moss et al., who demonstrated that in the eighth segment of the IAV, the splicing site is situated within a structure that can exist in an equilibrium between pseudoknot and hairpin formations (Figure 1) [14,19]. This can influence virus splicing.

Moreover, Jiang and colleagues showed that mutation, designed by ensemble defect, misfolded the secondary structure of segment 8 mRNA and affected IAV mRNA splicing and replication in cell cultures. The relative amount of NS2 mRNA was significantly lower for the IAV pseudoknot mutants than for the WT virus. At the same time, NS1 mRNA showed similar levels in WT and mutant viruses in MDCK 2 cells at 3 h post-infection at an MOI 5. Furthermore, the multi-cycle growth kinetics of IAV mutants and WT viruses showed attenuated replication kinetics in both MDCK 2 and A549 cell lines [14].

The pseudoknot, which contains the splicing site, can control splicing, but the knowledge how splicing is regulated is still limited. Here, we wanted to check whether PKR, in addition to its antiviral properties, can also influence the splicing of IAV mRNAs. For that, we used 2-AP, a PKR inhibitor, and analyzed the expression of NS1 and NS2. We expected that 2-AP treatment would increase viral titers or at least allow the virus to grow at the same level as the control. Surprisingly, we noticed that 2-AP inhibits virus replication in A549 (virus titer lower by about 1 log) and MDCK 2 (virus titer lower by about 2 log) cells. Only slight differences in viral titers were observed in HEK-293T cells (Figure 2). The discrepancies among the effects of 2-AP in cell lines can be connected with different interferon responses in used cell cultures. HEK-293T cells lack an intact immune response [20], so the addition of 2-AP is supposed to not change virus replication, as we see it. The difference between A549 and MDCK 2 cells can also be a result of an immune response. It was shown that A549 cells produce a high level of MxA protein in response to IFN, which can be responsible for lower virus titers [21].

Our results show that 2-AP can be an anti-IAV drug, but its antiviral effect is cell-dependent, and more studies are needed to elucidate its detailed mechanism of action. In addition, the effect of 2-AP may be virus-specific, since the addition of 2-AP to cell cultures pretreated with INF-γ restores the amount of the porcine reproductive and respiratory syndrome virus [22]. Moreover, to understand how 2-AP affects the splicing and replication of IAV, some *in vivo* experiments with laboratory animals with different IAV strains, including the IAV mutant described by Jiang et al. [14] would be interesting to check. In addition, experiments with genetically modified animals to express some crucial human factors, such as MxA, would be also beneficiary. Finally, the NS1 and NS2 expression and NS1/NS2 ratio should be analyzed in different animal tissues, where IAV may have different replication kinetics, i.e., due to the IFN-sensitivity. All of these above were out of the scope of this article.

To study if the inhibition of PKR affects influenza splicing, we checked the ratio of NS2 mRNA to NS1 mRNA (Figure 3). The literature has shown that the ratio of NS2 mRNA to NS1 mRNA is between 10 and 15% and is constant during infection [5,23,24]. This is in line with our studies for MDCK 2 cells, where the level of NS2 mRNA to NS1 mRNA was between 7.4 and 11.9%. The lower ratio was observed with the HEK-293T cells (3.9–5.9%). An interesting finding was made with A549 cells, wherein the control reaction ratio between those mRNAs was 6.6% but increased to 22.5% after 2-AP treatment. Explaining this observation requires additional experiments that are beyond this study. To summarize, it can be indicated that 2-AP has some influence on IAV mRNA splicing, but the effect is cell-dependent.

The results on NS1/NS2 mRNA expression are in agreement with the western blot analysis, where we checked NS1 and NS2 protein levels (Figure 4). HEK-293T cells indicated the same level of viral non-structural proteins in 2-AP and mock-treated cells. In contrast, in MDCK 2 and A549 cells, we observed significantly decreased levels of NS1 and no expression of NS2 in 2-AP-treated cells.

## 5 Conclusions

This study examined the impact of the PKR inhibitor, 2-AP, on IAV mRNA replication and splicing, particularly focusing on segment 8, which encodes the NS1 and NS2 proteins. We aimed to determine if 2-AP’s PKR inhibition could influence IAV mRNA splicing and viral replication.

The results showed that 2-AP significantly reduced viral replication in MDCK 2 and A549 cells, particularly in MDCK 2, where virus titers dropped by up to 98%. In contrast, HEK-293T cells, lacking a complete immune response, showed only a minor reduction, suggesting that 2-AP’s antiviral effect is linked to an intact immune response.

Unexpectedly, 2-AP demonstrated antiviral activity regardless of its PKR inhibition, indicating it may have previously unknown antiviral properties that vary by cell type. Our analysis revealed no significant changes in the NS2/NS1 splicing ratio in MDCK 2 or HEK-293T cells, but a marked increase in A549 cells treated with 2-AP. This suggests a unique influence on IAV mRNA splicing by 2-AP in specific cell lines.

In conclusion, while PKR inhibition does not directly alter IAV mRNA splicing as we hypothesized, 2-AP reduces viral replication and modifies splicing ratios in a cell-specific manner. Further research is necessary to understand the mechanisms behind these effects and to explore 2-AP’s potential as a novel antiviral agent against IAV.
